# Contents of total fat, fatty acids, starch, sugars and dietary fibre in Swedish market basket diets

**DOI:** 10.1017/S0007114515000501

**Published:** 2015-04-02

**Authors:** W. Becker, A. Eriksson, M. Haglund, S. Wretling

**Affiliations:** National Food Agency, PO Box 622, SE-75126Uppsala, Sweden

**Keywords:** Market baskets, Fat, Fatty acids, Starch, Sugars, Polyols, Dietary fibre, Sweden

## Abstract

The typical dietary supply of total fat, fatty acids, starch, sugars, polyols and dietary fibre in Sweden was assessed from analyses of market baskets (MB) purchased in 2005 and 2010. MB were based on food balance sheets, with each basket comprising about 130 foods, which represented more than 90 % of annual dietary supply. Foods were divided into ten to twelve categories. In 2010, total fat contributed 34 % of energy (E%), SFA 14·3 E%, MUFA 12·8 E%, PUFA 4·6 E%, *n*-6 fatty acids 3·6 E%, *n*-3 fatty acids 1·0 E% and *trans-*fatty acids (TFA) 0·5 E%. Glycaemic carbohydrates contributed 47 E%, monosaccharides 9 E%, sucrose 11 E%, disaccharides 15 E% and total sugars 24 E%. Added sugars contributed about 15 E%. Dietary fibre content was about 1·7 g/MJ in the 2010 MB. Compared with the 2005 MB, the dietary supply of TFA and dietary fibre was lower, otherwise differences were small. The present MB survey shows that the content of SFA and added sugars was higher than the current Nordic Nutrition Recommendations, while the content of PUFA and especially dietary fibre was lower. TFA levels decreased and dietary supply was well below the recommendations of the WHO. These results emphasise a focus on quality and food sources of fat and carbohydrates, limiting foods rich in SFA and added sugars and replacing them with foods rich in dietary fibre and *cis*-unsaturated fatty acids.

Data on dietary intakes of nutrients are generally obtained from dietary surveys using various assessment methods. The range of nutrients that can be assessed is limited by the available food composition database. Dietary carbohydrates comprise a range of constituents including starch, mono- and disaccharides, as well as polyols and dietary fibre. The coverage of these constituents of carbohydrates in food composition databases is often limited, leading to uncertainties in dietary exposure assessments from dietary surveys. The same applies for various fatty acids, e.g. *trans-*fatty acid (TFA) isomers and some long-chain PUFA. Other complementary approaches are market basket (MB) and total diet studies, which are used to estimate the average dietary exposure to various dietary components, especially minerals and contaminants^(^ ^1^ ^–^ ^4^ ^)^. The purpose of the present study was to describe the content of total fat, fatty acids and carbohydrate constituents in Sweden using analytical data from MB. The results of the analysis are compared with calculations that are based on Swedish food composition database and food consumption surveys. Previously, the MB approach has been used in Sweden to assess dietary exposure to essential mineral elements and contaminants such as heavy metals, halogenated hydrocarbons and radionuclides^(^ ^5^ ^–^ ^9^ ^)^.

## Materials and methods

### Market baskets

The choice of food items included in the MB was based on food balance sheets (FBS) that are managed by the Swedish Board of Agriculture (SBA). Food consumption data for the 2005 MB were based on the FBS for 2003^(^ ^10^ ^)^, and on the 2007 FBS for the 2010 MB^(^ ^11^ ^)^. FBS give information on annual market availability of food categories and foodstuffs. Supplementary purchase statistics for fish and fats (for 2009/2010) were obtained from the market research company Growth from Knowledge, Sweden. This is due to the lack of detailed data on fresh fish and fats in the SBA report. The Growth from Knowledge statistics are based on their consumer panels, and can be transformed into values representing the total consumption volume (in kg) and representing some of the leading products and specific types or products of fish.

A shopping list was produced by breaking down the food categories into food items using data for their market shares (see online Supplementary Table S1). Food categories were included based on their average consumption of 0·5 kg/person per year (i.e. 1·5 g/person per d) or more. The list covers approximately 90 % of the total annual consumption expressed in kg/person. For each food category, one or more individual food items were selected depending on the level of detail in the statistics. For food categories such as wheat flour, milk, butter, eggs, tomatoes and oranges, one sample was generally purchased. For food categories comprising mixed foods such as bread, pastries, sausages, fat spreads and oils, and vegetable and fruit preserves, several products/brands were purchased in relation to consumption. This means that each basket represents more than 130 food items. Foods excluded were coffee and tea, tap water, household salt and alcoholic beverages. Beer with < 3·5 vol% alcohol, which is available in regular food stores, was included.

In August–December 2005, food baskets were obtained from two major department stores in each of four larger Swedish cities (Malmö, Gothenburg, Uppsala and Sundsvall), representing different regions and major populations areas^(^ ^7^ ^,^ ^9^ ^)^. Thus, the total number of baskets was eight. Due to practical reasons and similar product/brand assortment, ice cream and fats were purchased in Uppsala only. An evaluation of the results from these surveys showed in most cases no significant and consistent differences between food baskets from these cities, and for the 2010 MB, food baskets were collected from Uppsala only. The Uppsala baskets were collected from five different major grocery chains (Coop, ICA, Willys, Hemköp and Lidl). The purchases were all made in May–June 2010, plus a supplementary purchase of fruit, vegetables and potatoes was made in the autumn of the same year (September–October) with the purpose of obtaining more Swedish-grown products. Due to the delay in obtaining consumption data on fish products, sampling of this food group was postponed and synchronised with vegetables (September–October). One objective of food sampling in 2010 was to examine the possible differences between standard-price and low-price products. Based on this approach two food baskets were collected at each food chain: one standard-price and one low-price basket. For one of the food chains (Lidl), only one basket was collected because of a limited selection of food items within each food group. A total of nine different food baskets were collected from these Uppsala food stores during spring 2010, and five supplementary purchases of vegetables, fruits and potatoes (of what was defined as being in the standard-price category) were made from these food chains in the autumn of the same year.

In 2005, staff from the local health authorities in each city made the purchases, except in Uppsala where staff from the National Food Agency (NFA) made the purchase. In the 2005 MB, each shopper was instructed to take advantage of price offers, since price has been shown to be a major factor for food choice^(^ ^12^ ^)^. In the 2010 MB, the focus was to compare normal-price with low-price alternatives. Immediately after the purchase, the baskets were transported to the NFA in Uppsala. In both studies, more than 1000 food items were purchased.

### Preparation of food samples

Food items in each basket were divided into twelve groups, of which ten were analysed for carbohydrates (Table 1). Grouping is based on the categorisation in the Swedish Food Circle (vegetables, fruit, potatoes, bread/cereals, dairy products, meat, fish, eggs and fats) in combination with categories defined in the SBA statistics (sweet bakery products, sugar/sweets and beverages). Food groups not contributing to carbohydrate intake (eggs, fats and oils) and fat (beverages) were excluded from the analysis. For each food group, a sample was prepared to represent 1 % of the average annual food consumption for an average person. The food items in each food group were treated as they would be in a general household; for example, meat was freed from bones, fresh fish were freed from skin and bones, and potatoes and root vegetables were peeled. The samples were then mixed and homogenised in an acid-washed food blender with a bowl of acrylonitrile styrene plastic and equipped with a titanium blade. The samples were stored in acid-washed containers at − 20°C until analysis. In the 2005 MB, homogenates from the two baskets purchased in each of the four cities were merged before the analysis, resulting in one sample per food group and city.

**Table 1:** Food groups included in market baskets purchased in four different cities in Sweden in 2005 and 2010 and weights of food homogenates (representing 1 % of annual per capita consumption)

Food groups	No. of food items (2005)*	No. of food items (2010)	Weight of food group homogenates (g) in 2010	Fatty acid factor	Description of food items included in the food group
Cereals	11	11	844	0·70	Flour, grains, breakfast cereals, pasta and bread
Pastries	5	5	185	0·95	Biscuits, buns and cakes
Meat	16	15	759	0·95	Beef, pork, lamb, poultry and cured/processed meats
Fish	14	16	185	0·90	Fresh and frozen, canned products and shellfish
Dairy products	16	18	1557	0·95	Milk, sour milk, yogurt, cream, hard cheese, processed cheese and cottage cheese
Eggs	–	1	84	0·83	Fresh eggs
Fats	–	13	145	0·956	Butter, spreads, cooking oil and mayonnaise
Vegetables, including root vegetables	19	19	704	0·80	Fresh and frozen, and canned products
Fruit and berries	15	18	867	0·80	Fresh and frozen, canned products, juice, nuts, cordials and jam
Potatoes	4	4	458	0·95	Fresh, French fries, mashed potato powder and crisps
Sweets, sugar, ice cream	9	11	453	0·95	Chocolate, sugar-based sweets, dairy and vegetable fat-based ice cream, sugar, mustard and ketchup
Beverages	4	5	1205	–	Soft drinks, mineral water and beer (*<*3·5 vol% alcohol)

* Represent a specific food item or category, e.g. white bread and herring. Several categories are mixed samples (two or more brands/types of the same category).

### Chemical analyses

#### Fat and fatty acids

##### 2010

Total fat was analysed in all food groups, except beverages (i.e. twenty-two samples), by accredited gravimetric standard methods. Fat in dairy products, fats, and sugar and sweets was analysed by the Röse–Gottlieb method according to the Nordic Committee on Food Analysis (NMKL)^(^ ^13^ ^)^, and in cereal products, pastries, meat, fish, eggs, vegetables, fruits and potatoes by the Schmidt–Bondzynski–Ratzlaff (SBR) method according to the NMKL^(^ ^14^ ^)^. Fatty acids were analysed in all food groups except beverages by an in-house validated and accredited method. Fat was extracted according to the method of Folch *et al.* ^(^ ^15^ ^)^. Fatty acids in the fat were converted to methyl esters and separated on a capillary column. Reference standards containing individual SFA, MUFA and PUFA were used for identification^(^ ^16^ ^)^. TFA were analysed according to an American Oil Chemists' Society standard method^(^ ^17^ ^)^ by GC using a 100 m HP-88 capillary column for separation. The limit of detection was 0·03 % for each fatty acid. Analyses were performed in March 2011 (total fat) and August 2011 (fatty acids).

##### 2005

Total fat in the homogenates of cereals, pastries, vegetables, potatoes and fruit was analysed as raw fat according to EC-directive 98/64/EC^(^ ^18^ ^)^. Total fat in dairy products and sweets was analysed by the Röse–Gottlieb method according to the NMKL^(^ ^19^ ^)^, and in meat, fish, eggs and fats according to the SBR-NMKL^(^ ^14^ ^)^. Fatty acids were analysed after extraction according to the method of Folch *et al.* ^(^ ^15^ ^)^. Fatty acids were converted to methyl esters and were separated on a capillary column. Reference standards containing individual SFA, MUFA and PUFA were used for identification of the fatty acids^(^ ^16^ ^)^. TFA were analysed using a 100 m capillary column. The laboratories are accredited for the use of the aforementioned methods. Analyses were performed during spring 2007. Total fat was analysed by the National Veterinary Institute, Uppsala, and fatty acids at NFA.

#### Carbohydrates

##### 2010

Sugars (glucose, fructose, sucrose, maltose and lactose) and starch were analysed in all food groups, except eggs and fats. Sugars were analysed by a GC method described elsewhere^(^ ^20^ ^)^. Starch was analysed by an enzymatic standard method according to the NMKL^(^ ^21^ ^)^. The methods for both sugars and starch were accredited for all food groups except fish at the time of the analysis. Validation for fish was done during the survey and accreditation for fish was received afterwards. Both methods had a limit of detection of 0·03 g/100 g. Dietary fibre was analysed in cereal products, pastries, vegetables, fruits and potatoes by an accredited enzymatic, gravimetric standard method according to the NMKL^(^ ^22^ ^)^. All samples were analysed during spring 2011.

##### 2005

Starch was determined enzymatically by a NMKL method^(^ ^21^ ^)^. Analyses were carried out in February 2008. Mono- and disaccharides (glucose, fructose, sucrose, lactose and maltose) and polyols (xylitol and sorbitol) were determined by GLC using an in-house, validated method^(^ ^20^ ^)^. Carbohydrates were converted to trimethylsilyl ethers after extraction with 80 % ethanol and analysed by GLC using flame ionisation detection, followed by quantification using a calibration curve with phenyl-β-d-glucoside as the internal standard. The analyses were carried out in November–December 2007. Total dietary fibre content was determined gravimetrically according to the Association of Official Analytical Chemists/NMKL^(^ ^22^ ^)^. Samples were treated with the enzymes Termamyl^®^, protease and amyloglucosidase, filtered, washed, dried and weighed. Total dietary fibre was then determined gravimetrically as the remainder after correction for protein and ash weight. The analyses were carried out in November–December 2006.

The analyses of starch, sugars and polyols were performed at NFA, while analyses of dietary fibre were done by Eurofins Food Agro, Lidköping.

### Analytical quality control

The laboratories are accredited for the use of the aforementioned methods.

### Calculation of daily supply

The average daily supply of individual nutritional components of each food group was calculated by multiplying the concentration by the amount representing daily consumption according to the statistics.

## Results

### Fat and fatty acids in the food groups

The concentrations of major fatty acid categories in the food groups are given in Table 2. The proportion of SFA was highest in dairy products, sugar and sweets, pastries, and meat. TFA concentrations were generally below 1 % of total fatty acids, with the exception of dairy products, meat and fats. The proportion of PUFA was highest in vegetables, cereal products and fish. The proportion of *n*-3 fatty acids was highest in fish, while the proportion of *n*-6 fatty acids (mainly linoleic acid) was highest in cereal products and vegetables.

**Table 2:** Content of total fat (g/100 g food) and major fatty acid categories (g/100 g fatty acids) in the food groups included in market baskets purchased in 2005 and 2010 (Mean values and ranges)

		Total fat	SFA	MUFA	PUFA	TFA	*n*-6	*n*-3
Food groups	Year	Mean	Range	Mean	Range	Mean	Range	Mean	Range	Mean	Range	Mean	Range	Mean	Range
Cereals	2005	2·6	1·7–3·4	18·1	15·1–22·0	38·7	33·0–46·2	43·0	38·5–49·0	0·2	0–0·6	37·3	31·9–44·7	5·7	4·3–6·6
	2010	2·2	2·2–2·2	19·0	16·9–21·0	39·7	38·6–40·8	41·2	40·1–42·3	0·37	0·34–0·39	36·0	35·2–36·8	5·2	4·9–5·5
Pastries	2005	17·9	16·4–19·4	48·8	43·8–53·8	36·6	33·5–40·4	14·6	12·0–17·1	2·7	0·6–6·4	11·8	9·9–13·6	2·8	2·0–3·5
	2010	20·0	19·6–20·4	46·7	45·2–48·3	39·1	37·9–40·3	14·0	13·6–14·4	0·78	0·66–0·89	11·8	11·5–12·0	2·1	2·0–2·2
Meat	2005	13·6	12·0–15·0	40·1	37·8–41·1	49·1	47·7–51·3	10·2	9·3–10·6	1·3	1·0–1·5	9·0	8·1–9·3	1·1	1·0–1·1
	2010	11·9	11·8–12·0	41·4	40·8–42·1	48·7	48·7–48·8	8·8	8·4–9·1	1·5	1·33–1·65	7·5	6·7–8·2	1·1	1·0–1·1
Fish	2005	8·8	6·6–10·0	16·9	16·2–17·7	46·4	44·5–48·2	35·0	34·3–36·2	1·1	0·8–1·6	16·9	14·6–18·7	17·4	16·3–18·4
	2010	11·5	10·9–12·1	15·8	15·5–16·1	49·9	49·7–50·0	33·9	33·8–34·0	0·82	0·80–0·83	16·5	15·9–17·1	16·6	16·2–17·1
Dairy products	2005	5·4	4·4–6·6	67·9	64·8–70·6	26·2	24·4–28·4	5·1	4·1–5·9	4·0	3·4–4·7	3·8	3·2–4·4	0·7	0·5–0·9
	2010	5·1	5·0–5·1	66·5	66·4–66·5	26·8	26·6–27·1	4·1	4·0–4·2	4·2	4·1–4·2	2·3	2·3–2·4	0·6	0·6–0·7
Eggs	2005	7·9	7·0–9·3	32·1	31·2–33·1	51·4	50·3–52·9	16·2	15·1–17·9	0·8	0·8–1·0	14·8	13·9–16·3	1·4	1·1–1·7
	2010	9·5	9·4–9·5	32·8	32·7–33·0	49·5	49·3–49·7	17·5	17·4–17·5	0·27	0·25–0·29	15·2	15·1–15·3	2·3	2·1–2·4
Fats	2005	67·3	37·2	43·3	19·5	0·9	14·4	5·0
	2010	66·7	38·0	41·8	19·7	1·19	14·9	4·5
Vegetables, roots	2005	0·4	0·2–0·5	27·0	26·1–27·6	13·7	11·9–18·1	57·3	52·2–59·7	0·4	0·4–0·5	43·9	40·4–45·2	13·4	11·8–14·8
	2010	0·2	0·2–0·2	27·1	25·9–28·4	16·0	14·9–17·2	54·9	53·0–56·9	0·46	0·44–0·48	42·7	41·1–41·4	12·1	11·4–12·7
Fruit and berries	2005	0·6	0·5–0·6	11·4	10·7–12·6	72·0	65·9–74·7	14·9	13·0–18·0	< 0·1	0–0·3	13·5	11·7–16·1	1·4	1·2–1·9
	2010	1·05	0·9–1·2	10·1	9·9–10·3	77·3	77·1–77·5	11·7	11·5–12·0	–	10·9	10·6–11·1	0·9	0·88–0·91
Potatoes	2005	2·5	2·0–2·8	47·7	45·0–49·5	42·0	41·0–44·6	10·2	9·5–11·2	0·2	0–0·4	9·9	9·2–10·9	0·3	0·3–0·4
	2010	1·9	1·7–2·1	30·9	26·9–34·8	58·8	57·2–60·3	10·2	7·8–12·5	0·3	0·30–0·38	9·6	7·4–11·8	0·4	0·23–0·61
Sweets, ice cream, sugar	2005	2·9	2·1–4·1	60·3	53·8–63·3	33·2	31·8–37·1	6·3	4·6–9·2	0·3	0–0·8	5·7	4·2–8·5	0·5	0·4–0·7
	2010	11·8	11·5–12·1	50·6	50·5–50·8	38·6	38·1–39·1	10·8	10·5–11·0	0·5	0·46–0·49	8·4	8·0–8·9	2·2	1·9–2·5

TFA, *trans*-fatty acids.

There were generally small differences between the 2005 and 2010 MB, although TFA levels in pastries were lower in 2010 than in 2005 (Table 2). The fat content in the fish group was higher, which can be mainly attributed to a large proportion of salmon. In the 2005 MB, variation between the cities was generally moderate, with CV being 10–20 % or less. Large ranges were observed for TFA, for example, in pastries. Of the sixty fatty acids included in the standard assay, only a few were not detected in each MB (15 : 1, 16 : 0 anteiso, 18 : 0 anteiso, 22 : 2*n*-6, 22 : 4*n*-3, 22 : 5*n*-6 and 23 : 0). Positional isomers of unsaturated fatty acids were not specified further.

### Carbohydrates in the food groups

The concentrations of carbohydrate constituents in the food groups are given in Table 3. Starch content was highest in cereals, followed by potatoes and pastries. The content of glucose and fructose was high in fruit and berries, while the content of sucrose was highest in sugars and sweets, pastries, and soft drinks. Maltose was mainly found in cereals. Polyols were analysed in the 2005 MB and sorbitol was detected in small amounts in fruit and berries (jam and cordials) and in sweets. Xylitol was not detected in any of the food groups. The content of dietary fibre was highest in cereals. There were generally small differences between the 2005 and 2010 MB, although sucrose content in fruit and berries was lower in 2005 than in 2010 (Table 3). In the 2005 MB, variation between the cities was generally low or moderate (CV < 10–20 %) for major constituents (>2 g/100 g) in the various food categories. In the 2010 MB, differences in the concentrations of fatty acid categories and carbohydrate constituents in the food groups between the standard-price and low-price baskets were generally small.

**Table 3:** Content of carbohydrate constituents (g/100 g) in the food groups included in market baskets purchased in Sweden in 2005 and 2010 (Mean values and ranges)

Food groups	Starch	Dietary fibre	Fructose	Glucose	Sucrose	Lactose	Maltose	Sorbitol
Mean	Range	Mean	Range	Mean	Range	Mean	Range	Mean	Range	Mean	Range	Mean	Range	Mean	Range
Cereals																
2005	48·4	47·5–49·9	5·5	4·9–6·0	1·2	0·7–1·8	1·2	1·0–1·7	0·3	0·29–0·47	0·2	0·20–0·32	2·6	2·1–3·2	ND
2010	46·8	45·8–47·7	4·1	3·1–5·0	1·3	1·2–1·3	1·1	1·1–1·2	0·3	0·29–0·36	0·2	0·21–0·21	2·0	2·0–2·1	NA
Pastries																
2005	25·0	22·3–26·2	2·9	2·6–3·2	1·4	1·1–2·0	2·2	1·8–2·9	19·3	18·3–20·3	0·3	0–0·83	1·1	0·74–1·8	ND
2010	25·0	23·9–26·1	2·5	2·3–2·7	1·1	1·0–1·1	1·4	1·2–1·5	21·7	20·2–23·1	ND	0·8	0·69–0·83	NA
Dairy																
2005	ND	NA	< 0·1	0·2	0·16–0·25	0·25	0·19–0·33	3·6	3·4–3·9	NA	ND
2010	ND	NA	ND	0·1	0·12–0·14	0·4	0·38–0·41	3·5	3·2–3·8	NA	NA
Meat																
2005	1·3	1·1–1·6	< 1	0·1	0·07–0·12	0·6	0·51–0·62	0·3	0·24–0·35	0·2	0·12–0·36	0·2	0·16–0·18	ND
2010	1·3	1·0–1·6	NA	< 0·1	0·6	0·57–0·64	0·2	0·16–0·21	0·1	0·03–0·16	0·4	0·28–0·42	NA
Fish																
2005	1·4	1·2–1·4	NA	< 0·1	0·3	0·18–0·42	2·3	2·1–2·6	0·1	0·12–0·15	0·2	0·08–0·30	ND
2010	1·6	1·5–1·6	NA	< 0·1	0·2	0·14–0·17	2·0	1·9–2·1	< 0·1	0·3	0·22–0·29	NA
Vegetables																
2005	0·3	0·24–0·36	2·0	1·8–2·4	1·8	1·6–1·9	1·8	1·8–1·9	ND	ND	0·1	0·09–0·11	ND
2010	0·4	0·33–0·51	1·9	1·7–2·1	2·1	2·0–2·3	1·8	1·8–1·9	ND	ND	0·1	0·10–0·12	NA
Fruit and berries															
2005	0·5	< 0·05–0·90	1·7	1·5–1·9	8·8	7·7–10·2	8·7	7·5–9·4	1·9	1·2–2·7	ND	0·4	0·20–0·58	0·5	0·41–0·61
2010	0·2	ND–0·45	1·7	1·6–1·7	7·4	6·8–8·1	6·3	5·8–6·8	5·2	5·1–5·4	ND	0·2	0·14–0·22	–
Potatoes																
2005	17·5	15·0–19·9	2·4	2·1–2·9	0·5	0·37–0·51	0·6	0·52–0·68	< 0·1		ND	0·1	0·08–0·19	ND
2010	15·8	15·7–15·8	2·2	1·9–2·4	0·3	0·20–0·48	0·4	0·29–0·46	0·2	0·05–0·26	ND	0·1	0·11–0·13	NA
Sweets, ice cream															
2005	4·8	3·7–7·1	NA	1·8	1·1–2·6	4·2	3·7–5·0	48·3	47·8–48·6	2·6	2·4–2·8	2·5	2·2–3·0	0·1	ND–0·29
2010	3·0	2·5–3·5	NA	2·0	2·0–2·1	3·8	3·5–4·1	38·1	37·6–38·6	2·1	2·0–2·2	1·1	1·0–1·1	NA
Soft drinks, beer															
2005	NA	NA	0·9	0·56–1·2	1·1	0·65–1·4	3·1	1·5–3·7	ND	ND	ND
2010	NA	NA	1·1		0·94		4·1		ND	ND	NA

ND, not detected ( < 0·05 g/100 g); NA, not analysed.

### Daily supply

**Fig. 1:**
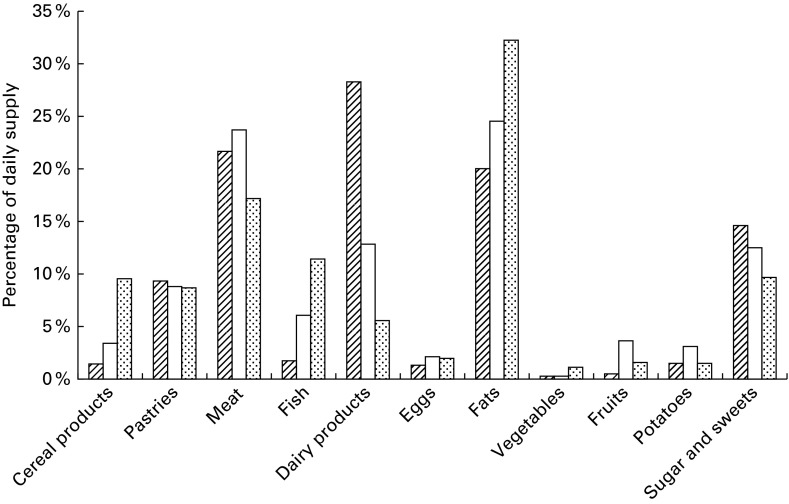
Percentage contribution of major fatty acid categories from the food groups included in market baskets purchased in 2010. 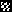, SFA; 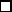, MUFA; 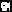, PUFA.

**Fig. 2:**
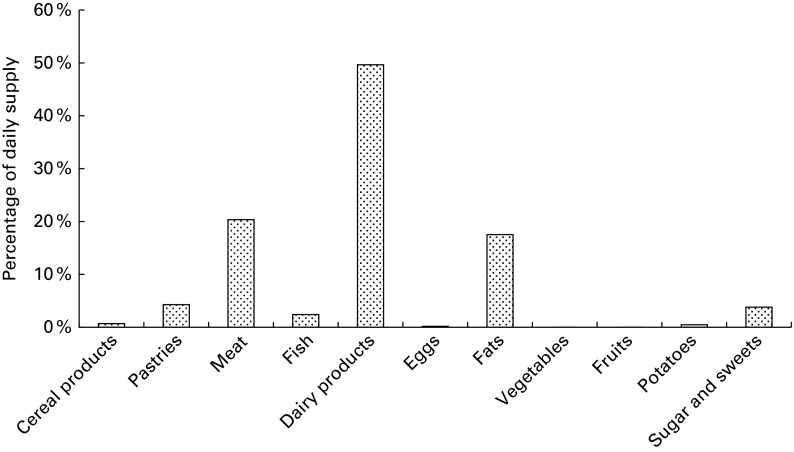
Percentage contribution of *trans-*fatty acids from the food groups included in market baskets purchased in 2010.

**Fig. 3:**
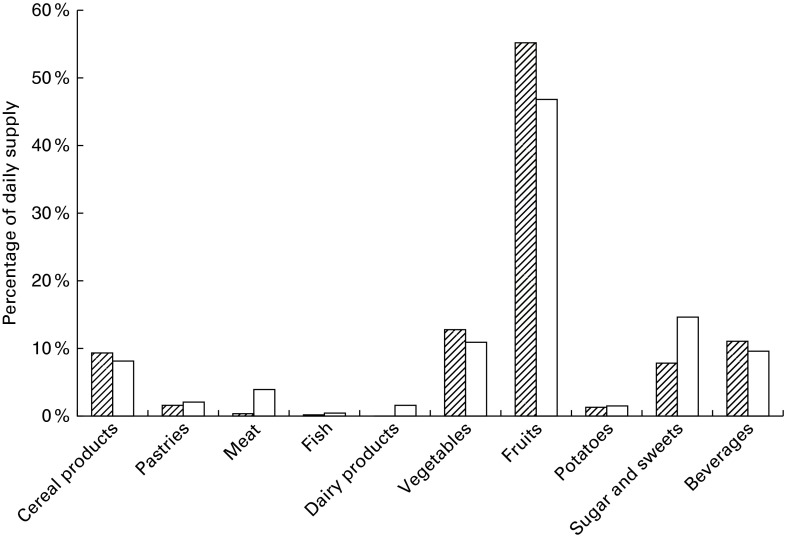
Percentage contribution of monosaccharides from the food groups included in market baskets purchased in 2010. 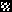, Fructose; 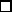, glucose.

**Fig. 4:**
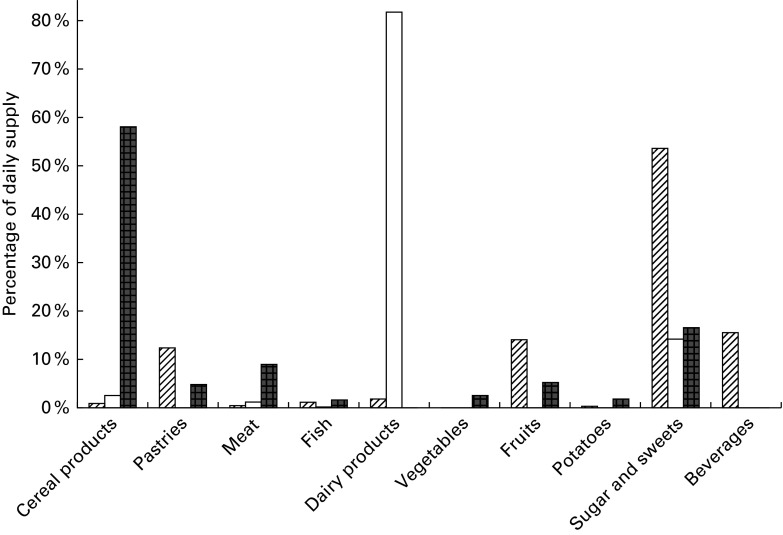
Percentage contribution of disaccharides from the food groups included in market baskets purchased in 2010. 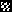, Sucrose; 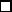, lactose; 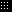, maltose.

**Fig. 5:**
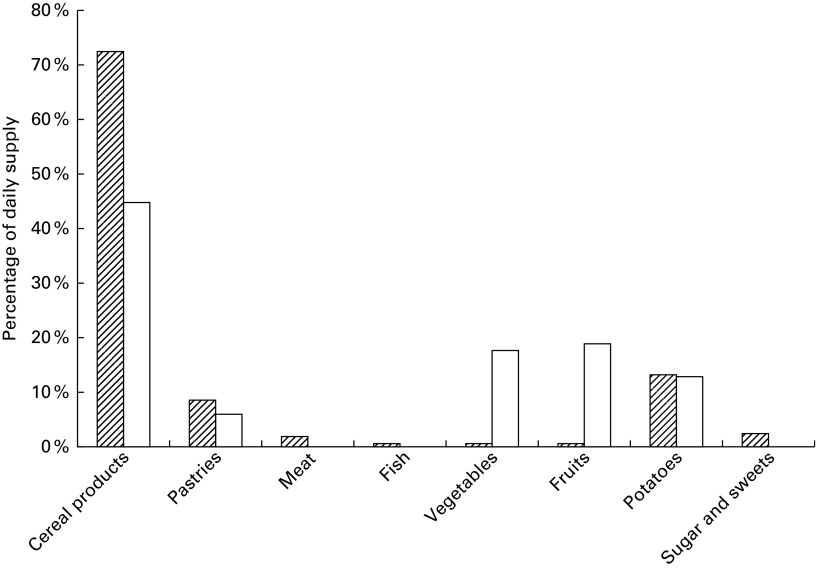
Percentage contribution of starch and dietary fibre from the food groups included in market baskets purchased in 2010. 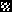, Starch; 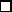, fibre.

The average daily dietary supply of total fat and fatty acids and of carbohydrate constituents are given in Tables 4 and 5. Percentage contribution from food groups in the 2010 MB is shown in Figs. 1–5. The content of glycaemic carbohydrates was calculated as the sum of starch and total sugars. The term ‘glycaemic carbohydrates’ is defined as carbohydrates that are absorbed in the small intestine and includes oligosaccharides, in addition to starch and sugars^(^ ^23^ ^)^.

**Table 4:** Content of total fat and major fatty acid categories (g/person per d) included in market baskets purchased in 2005 and 2010 (Means values and ranges)

Food groups	Year	Total fat	SFA	MUFA	PUFA	*Trans*-fatty acids	*n*-6 Fatty acids	*n*-3 Fatty acids
Cereals	2005	6·5	0·8	1·8	1·9	0·01	1·66	0·27
	2010	5·1	0·68	1·4	1·5	0·01	1·28	0·18
Pastries	2005	9·4	4·3	3·2	1·3	0·24	1·04	0·25
	2010	10·1	4·5	3·8	1·35	0·07	1·14	0·20
Meat	2005	26·4	10·8	11·5	2·6	0·33	2·3	0·31
	2010	24·8	10·5	10·2	2·66	0·35	2·30	0·36
Fish	2005	4·1	0·6	1·7	1·3	0·04	0·63	0·64
	2010	5·8	0·82	2·6	1·8	0·04	0·87	0·86
Dairy products	2005	25·6	16·4	6·3	1·1	0·97	0·92	0·16
	2010	21·6	13·7	5·5	0·85	0·86	0·72	0·13
Eggs	2005	1·7	0·5	0·7	0·2	0·01	0·21	0·02
	2010	2·2	0·6	0·9	0·3	0·00	0·27	0·04
Fats	2005	26·6	9·4	11·0	4·9	0·23	3·6	1·3
	2010	26·5	9·6	10·6	5·0	0·30	3·9	1·1
Vegetables	2005	0·6	0·1	0·1	0·3	0·00	0·21	0·06
	2010	0·39	0·08	0·05	0·17	0	0·13	0·04
Fruit and berries	2005	1·0	0·1	0·6	0·1	0	0·11	0·01
	2010	2·5	0·2	1·5	0·2	0	0·2	0·02
Potatoes	2005	3·0	1·4	1·2	0·3	0·01	0·28	0·01
	2010	2·4	0·7	1·3	0·22	0·01	0·22	0·01
Sweets, ice cream	2005	2·8	1·7	1·0	0·2	0·01	0·16	0·01
	2010	14·6	7·0	5·36	1·50	0·07	1·19	0·31
Total (per person per d)	2005							
	Mean	108	46·2	39·1	14·2	1·9	11·2	3·0
	Range	102–116	42·4–49·8	35·3–42·4	13·1–14·9	1·5–2·2	10·4–11·8	2·7–3·2
	2010							
	Mean	116	48·3	42·1	15·3	1·7	12·0	3·3
	Range	115–117	48·0–48·5	40·1–44·2	14·6–16·1	1·7–1·8	11·6–12·5	3·1–3·5

**Table 5:** Content of starch, sugars, sorbitol and dietary fibre (g/person per d) included in market baskets purchased in 2005 and 2010 (Mean values)

	Year	Starch	Fructose	Glucose	Sucrose	Lactose	Maltose	Sorbitol	Glycaemic carbohydrates	Dietary fibre
Cereals	2005	121	3·0	3·0	0·9	0·6	6·6	ND	135	13·7
	2010	108·1	3·0	2·6	0·75	0·49	4·7	NA	120	9·4
Pastries	2005	12·9	0·8	1·2	10·0	< 0·1	0·6	ND	25·4	1·5
	2010	12·7	0·53	0·7	11·0	0	0·4	NA	25·2	1·3
Meat	2005	2·6	0·2	1·1	0·6	0·4	0·3	ND	5·2	NA
	2010	2·7	0·1	1·3	0·4	0·20	0·7	NA	5·4	NA
Fish	2005	0·6	< 0·1	0·2	1·1	0·1	0·1	ND	2·1	NA
	2010	0·8	< 0·1	< 0·1	1·01	< 0·1	0·1	NA	2·1	NA
Dairy products	2005	NA	0·1	1·0	1·2	17·2	ND	ND	19·5	NA
	2010	NA	0	0·55	1·7	15·1	ND	NA	17·3	NA
Vegetables, roots	2005	0·5	3·0	3·2	ND	ND	0·2	ND	6·9	3·5
	2010	0·8	4·1	3·5	0	0	0·2	NA	8·6	3·7
Fruit and berries	2005	1·0	16·5	16·3	3·5	ND	0·7	0·9	38·9	3·2
	2010	0·54	17·6	15·0	12·4	0·00	0·4	NA	46·0	3·9
Potatoes	2005	21·2	0·6	0·7	< 0·1	ND	0·1	ND	22·7	2·9
	2010	19·8	0·4	0·5	0·2	0	0·2	NA	21·0	2·7
Sweets, ice cream	2005	4·5	1·7	4·0	46	2·5	2·4	0·1	61·3	NA
	2010	3·7	2·5	4·7	47·3	2·6	1·3	NA	62·2	NA
Soft drinks, beer	2005	NA	3·2	3·7	10·7	ND	ND	ND	17·6	NA
	2010	NA	3·6	3·1	13·6	0·00	0·00	NA	20·3	NA
Total (per person per d)	2005	164	29·1	34·4	73·9	20·7	11·0	1·1	334	24·8
	2010	149·1	31·9	32·0	88·4	18·4	8·1	NA	328	20·9

ND, not detected; NA, not analysed.

#### Fat and fatty acids

The average dietary supply of total fat in the 2010 MB was 116 g/person per d. The major contributors were fats and oils (23 %), meat (21 %) and milk products (19 %). Pastries contributed 9 %, and sugar and sweets contributed 13 %. The average content of SFA was 48 g/person per d. Dairy products contributed 28 % SFA, meat 22 % SFA and fats 20 % SFA (Fig. 1). The average content of MUFA was 42 g/person per d, and the major contributors were meat (24 %) and fats (25 %), dairy products, and sugar and sweets, with each contributing 13 % (Fig. 1). The average content of PUFA was 15 g/person per d, of which 12 g was from *n*-6 fatty acids and 3·3 g from *n*-3 fatty acids, respectively. The major contributors of *n*-6 fatty acids (linoleic acid) were fats (32 %) and pastries (19 %). Fats contributed 35 % of *n*-3 fatty acids (as α-linolenic acid) and fish 26 %, mainly as EPA and DHA. The average exposure to TFA was 1·7 g/person per d. The major contributors were dairy products (50 %), followed by meat (20 %) and fats (18 %). Dairy products also contributed the main part of the individual TFA isomers (Fig. 2).

The average dietary supply of individual fatty acids is shown in online Supplementary Table S2. Palmitic acid (16 : 0) was the main SFA followed by stearic acid (18 : 0) and myristic acid (14 : 0). Oleic acid (18 : 1) was the main MUFA, while linoleic acid was the main PUFA, followed by α-linolenic acid. Long-chain *n*-3 fatty acids, EPA and DHA, contributed 0·18 and 0·33 g/person per d, respectively. 18 : 1*t* was the main *trans* isomer.

Compared with the 2005 MB, the dietary supply of total fat was higher, mainly due to a large contribution from the sugar and sweets group, in which chocolate and ice cream are high in fat. This difference may be due to the fact that ice cream was under-represented in the 2005 MB. The content of TFA was 1·7 g/person per d, compared with 1·9 g/d in 2005. A major decrease in TFA content was observed in the pastries group, which in 2005 contributed 13 % of the total TFA content, compared with 4 % in 2010.

#### Carbohydrates

In the 2010 MB, the average dietary supply for both glucose and fructose was 32 g/person per d, of which fruits contributed about half, and each of the food groups cereal products, vegetables, sugar and sweets, and beverages contributed about 10 % (Table 5 and Fig. 3). The dietary supply of sucrose was 88 g/person per d, of which sugar and sweets contributed 54 %, while pastries and beverages each contributed 14–15 % (Fig. 4). Lactose content was 18 g/person per d, of which dairy products contributed on average 83 %, and sugar and sweets another 12 %. The supply of maltose was 8·1 g/person per d, cereal products contributing about 60 %, and sugar and sweets contributing an additional 22 %. The supply of starch was 149 g/person per d, of which cereal products contributed three-quarters and potatoes 13 % (Fig. 5). The supply of dietary fibre was 21 g/person per d, cereals contributing about half, vegetables and fruits contributing about one-fifth each, and potatoes 13 % (Fig. 5). The main contributors of glycaemic carbohydrates were cereals (37 %), sugar and sweets (19 %), and fruits, including jam and cordials (14 %).

Compared with the results of the 2005 MB, the average dietary supply of starch and dietary fibre was lower, while that of sucrose was higher (Table 5). In the 2005 MB, sorbitol was detected in the fruit and berries group, including fruit-based cordials and jam, and in the sugar and sweets group. Xylitol was not detected in any of the food groups.

## Discussion

The present study provides the most extensive analytical investigation to date of the composition of fat and carbohydrate constituents in the Swedish diet. It gives detailed data on more than fifty individual fatty acids and major carbohydrate components including mono- and disaccharides, starch, total dietary fibre and some polyols. In Sweden, two sets of FBS data are calculated^(^ ^11^ ^)^. In the present study, the data refer to the so-called ‘direct consumption’, which is relatively detailed and represents foods available for consumption at the retail and wholesale levels for an average person during a year. The amounts of food in the MB overestimate actual consumption, since edible losses due to waste in the retail and household sectors are not accounted for and because of some uncertainties in basic statistics. However, the data give an overall picture of the composition of the average diet, although the MB approach gives no information on food intake at the individual level. Foods vary in composition depending on variety, brand, season, etc. To increase variability, MB were purchased in two major food stores in four cities of Sweden in 2005. In 2010, two food baskets were purchased from each of four major food chains, one standard-price and one low-price basket, with complementary baskets from a fifth chain that did not cover the full list of included items.

The estimated energy supply in the 2010 MB was about 12·5 MJ/person per d, which is in line with calculations based on the total per capita supply (excluding energy from alcoholic beverages)^(^ ^11^ ^)^. Using this estimate, total fat was found to contribute 34 % of energy (E%), SFA 14·3 E%, MUFA 12·8 E%, PUFA 4·6 E%, *n*-6 fatty acids 3·6 E%, *n*-3 fatty acids 1·0 E% and TFA 0·5 E% in 2010. According to the Nordic Nutrition Recommendations 2012, intake of SFA should be limited to less than 10 E%, while intake of PUFA should be 5–10 E%, of which intake of *n*-3 fatty acids should be 1 E%^(^ ^24^ ^)^. Thus, the estimated supply of SFA was higher, while that of PUFA is slightly lower than the recommended lower threshold. Data from a recent national food consumption survey on adults carried out in 2010–11^(^ ^25^ ^)^ showed similar results for total fat (34 E%) and MUFA (12·8 E%), while the content of SFA (13·1 E%) was lower and that of total PUFA, *n*-3 and *n*-6 fatty acids was higher (5·6, 1·3 and 4·2 E%, respectively).

Some differences between the two MB were observed. The dietary supply of total fat was higher in the 2010 MB, mainly due to a large contribution from the sugar and sweets group, in which chocolate and ice cream have high fat content. The observed difference may be due to the fact that ice cream was under-represented in the 2005 MB. The supply of TFA was 1·7 g/person per d, compared with 1·9 g/person per d in 2005. A major decrease in TFA content was observed in the pastries group, which contributed 13 % of the total TFA content in 2005, compared with 4 % in 2010. TFA content corresponds to about 0·5 E%, which is well below the WHO recommendation stating that TFA should contribute with no more than 1 E%^(^ ^26^ ^)^. About 75 % of TFA are derived from ruminant sources.

Using the estimated energy supply of 12·5 MJ in the 2010 MB, monosaccharides contributed 9 E%, sucrose 12 E%, disaccharides 16 E% and total sugars 24 E%. Glycaemic carbohydrates contributed 45 E%. Dietary fibre content corresponded to approximately 1·7 g/MJ. The amount of added sugars was estimated from the content of monosaccharides and sucrose in the food groups. Monosaccharides and sucrose from all food groups, except for fruit and berries, jam and cordials, and potatoes, were calculated as added. Monosaccharides and sucrose in jam and cordials were also included, after correction for naturally occurring sugars in the fruit and berries group. The calculated amount of added sugars was 113 g/person per d, corresponding to approximately 15 E%. The corresponding calculations for the 2005 MB give similar estimates for energy distribution expressed in E%. The estimated supply of added sugars was higher than the upper limit of 10 E% according to the Nordic Nutrition Recommendations^(^ ^24^ ^)^.

In the dietary survey of children in Sweden in 2003, the calculated contribution of added sugars was 13–14 E%^(^ ^27^ ^)^. In a recent dietary survey on adults^(^ ^25^ ^)^, estimates were lower by about 10 E%, as was the intake of both monosaccharides (6·4 E%) and sucrose (7·7 E%). The content of dietary fibre was higher (2·0 g/MJ) in the 2005 MB than that (1·7 g/MJ) in the 2010 MB, which was lower than the recommended level of at least 3 g/MJ^(^ ^24^ ^)^. In recent Swedish dietary surveys on children and adults^(^ ^25^ ^,^ ^27^ ^)^, fibre intake was on average 1·7–1·8 and 2·5 g/MJ, respectively.

The results from the analyses were compared with calculations based on data in the NFA food composition database (version 04.1.1). There was generally a good agreement between estimates based on the MB and NFA food composition database, with differences within 5 % for major fatty acid categories. Analytical data gave a 28 % higher estimate of monosaccharide content and a 23 % lower estimate of sucrose and a 21 % lower estimate for dietary fibre content. However, both datasets gave a similar estimate for the content of total sugars. The content of glycaemic carbohydrates calculated from the analysis was about 45 g (14 %) lower than the calculated content of ‘available carbohydrates’ in the NFA food composition database. Values for ‘available carbohydrates’ in the database were calculated from the amount of total carbohydrates calculated ‘by difference’ after subtraction of dietary fibre content. However, values for ‘total carbohydrates’ calculated by difference may also include other non-carbohydrate components such as organic acids in fruit and vegetables. Thus, the data are not directly comparable.

MB studies or total diet studies investigating the content of fatty acids for other populations are relatively scarce, and mainly cover the period before the year 2000. A classic study has analysed the fatty acid composition of the Greenland Eskimo food in 1970s^(^ ^28^ ^)^, showing a high proportion and intake of long-chain *n*-3 fatty acids. More recent studies include total diet studies of Dutch^(^ ^29^ ^,^ ^30^ ^)^ and Finnish^(^ ^31^ ^)^ diets, analysis of duplicate diets in the seven-country study^(^ ^32^ ^)^, and studies evaluating dietary assessment methods^(^ ^33^ ^–^ ^35^ ^)^ or food composition tables^(^ ^36^ ^)^. The intake of TFA in several European countries during 1995–97 was assessed in the TRANSFAIR study using data from national dietary surveys and analytical data for TFA in foods^(^ ^37^ ^)^. Highest intakes were found to be 2·1 E% for men (Iceland) and 1·6 E% for women (The Netherlands), while lowest intakes were found to be 0·5 E% for men (Italy) and 0·8 E% for women (Greece). Recent data showed that TFA intakes decreased markedly in North European countries and ranged from 0·5 to 0·8 E%^(^ ^38^ ^–^ ^41^ ^)^.

A comparison of previous calculations of fat and fatty acid supply in Sweden based on FBS data^(^ ^42^ ^–^ ^44^ ^)^ indicated that the proportion of SFA decreased from the mid-1960s until the beginning of the 1990s, with a corresponding increase in MUFA and PUFA (Table 6). Since then, available data have indicated relatively minor changes. The corresponding calculations for TFA showed that the dietary supply was about 7 g/person per d in 1984 and about 3 g/person per d (1 E%) in 1994–95^(^ ^45^ ^)^. The results from the present MB study show that the dietary supply has decreased considerably since the mid-1990s. Åkesson *et al.* ^(^ ^46^ ^)^ analysed the content of 18 : 1 *trans* isomers in duplicate portions collected between 1968 and 1975 from twenty adults eating a mixed diet. Intake of *trans* isomers contributed about 2 E%. The analysis of duplicate portions from lacto-vegetarians (six subjects) and vegans (six subjects) collected between 1978 and 1980 in a health resort showed that the content of 18 : 1 *trans* isomers contributed about 1·3 and 0·5 E%, respectively^(^ ^47^ ^)^. In the present MB survey, 18 : 1 *trans* isomers contributed 0·4 E% in 2005 and 0·3 E% in 2010.

**Table 6:** Major fatty acid categories according to food disappearance statistics*

Year	SFA	MUFA	PUFA	Reference
1965	54	38	9·2	Becker^(^ ^43^ ^)^
1975	49	37	14	Bruce & Westin^(^ ^42^ ^)^
1980	47	37	15	Becker^(^ ^43^ ^)^
1992	45	39	16	Becker & Robertson^(^ ^44^ ^)^
2005 MB	46	40	14	Present study
2010 MB	45	340	15	Present study

MB, market basket.

* Percentage of total fatty acids. Values for 1965–92 were calculated from food composition data.

Calculations from dietary surveys on Swedish adults showed that intake of TFA was about 1 E% during the late 1990s^(^ ^37^ ^)^. The average intake of TFA among Swedish children in 2003 was 0·9 E%^(^ ^27^ ^)^. Since then, TFA content has decreased in several foods^(^ ^48^ ^)^, which can explain the lower level found in the MB studies. The results also showed that the average content of TFA was at the same level as in Denmark^(^ ^38^ ^)^, Norway^(^ ^40^ ^)^ and Finland^(^ ^41^ ^)^.

There are also some studies providing analytical data on the content of carbohydrates in the Swedish diet, dating from the 1970s. These include duplicate portion studies of twenty adults (25–60 years) and thirty-seven pensioners (67 years)^(^ ^49^ ^,^ ^50^ ^)^, lacto-vegetarian diets^(^ ^47^ ^)^ and vegan diets^(^ ^47^ ^,^ ^51^ ^)^. These results are shown in Table 7. Due to the limited number of subjects, being residents of a small community of South Sweden, no firm conclusions could be drawn with respect to time trends.

**Table 7:** Analytical data on carbohydrate constituents (g/10 MJ) according to the 2005 and 2010 market basket studies and previous duplicate diet studies in Sweden

	Sucrose	Glucose	Fructose	Maltose	Lactose	Sugars (total)	Starch
Market basket 2010	70·7	25·6	25·5	6·5	14·7	143	119
Market basket 2005	63	29	24	9	14	139	137
Adults, Dalby 1975^(^ ^49^ ^)^	50	16	NR	NR	17	NR	113
Elderly, Dalby 1979^(^ ^50^ ^)^	51	18	NR	NR	25	NR	113
Lacto-vegetarian diets, 1984^(^ ^47^ ^)^	19	38	42	9	17	126	112
Vegan diets, 1981^(^ ^51^ ^)^	49	NR	NR	NR	NR	NR	NR

NR, not reported.

MB studies or total diet studies from other countries or regions on carbohydrate constituents are also scarce and old. van Dokkum *et al.* ^(^ ^29^ ^)^ reported the content of carbohydrate constituents in the MB of male Dutch adolescents, while other studies have used the duplicate portion technique to validate food composition databases^(^ ^34^ ^)^ or dietary assessment methods^(^ ^52^ ^)^.

### Conclusions

The present MB survey shows that the dietary supply of SFA and added sugars in Sweden is higher than the current Nordic Nutrition Recommendations^(^ ^24^ ^)^, while the dietary supply of total PUFA and especially dietary fibre is lower. The results also indicate a need for updating of values for individual sugars in the database. The results are generally in line with recent national dietary surveys, and emphasise a focus on quality and food sources of fat and carbohydrates, replacing foods high in SFA and added sugars with foods that contribute with unsaturated fatty acids and naturally occurring dietary fibre.

## Supplementary material

For supplementary material accompanying this paper visit http://dx.doi.org/10.1017/S0007114515000501. click here to view supplementary material
