# Supplementary material for: Contents of total fat, fatty acids, starch, sugars and dietary fibre in Swedish market basket diets
Source: Br J Nutr. 2015 Apr 2;113(9):1453–65. doi: 10.1017/S0007114515000501 (PMC4462339; doi:10.1017/S0007114515000501)
Supplement: Supplementary file 1 [file S0007114515000501sup001.docx]

Web Table 1. Shopping list for the 2010 MB

| **Food group** | **Cate-gory** | **Selected food items**  Weighting factors within category (%) | **Comment** | **kg/L/yr** | **Purchase qnt.** | **Sample qnt., g** | **Waste, %** | **Weighed qnt., g** |
| --- | --- | --- | --- | --- | --- | --- | --- | --- |
|  |  | **Cereals** |  |  |  |  |  |  |
| 1 | 1 | Wheat flour |  | 6.9 | 2 kg | 69 | 0 | 69 |
| 1 | 3 | Rye flour, sifted |  | 0.5 | 2 kg | 5 | 0 | 5 |
| 1 | 4 | Rice, polished |  | 5.6 | 0.5-1 kg | 56 | 0 | 56 |
| 1 | 5 | Rolled oats |  | 3.4 | 1-1.5 kg | 34 | 0 | 34 |
| 1 | 8 | Gruel powder | 1 package | 0.8 | 2.5-5 L | 8 | 0 | 8 |
| 1 | 9 | Spaghetti/macaroni |  | 9.7 | 1 kg | 97 | 0 | 97 |
| 1 | 10 | Corn Flakes |  | 3.7 | 0.5 kg | 37 | 0 | 37 |
| 1 | 12 | Crisp bread, rye |  | 3.5 | 275-740 g | 35 | 0 | 35 |
| 1 | 14 | White bread (40%) | 1 loaf | 50.3 | ~0.6 kg | 201 | 0 | 201 |
| 1 | 14 | Bread, sifted rye (40%) | 1 loaf | 50.3 | ~0.5 kg | 201 | 0 | 201 |
| 1 | 14 | Rye bread (20%) | 1 loaf | 50.3 | ~0.7 kg | 101 | 0 | 101 |
|  |  |  |  |  |  |  |  | *844* |
|  |  | **Pastries** |  |  |  |  |  |  |
| 2 | 15 | Biscuits | Mixed biscuits | 5.0 | 300-600 g | 50 | 0 | 50 |
| 2 | 16 | Sweet wheat bread (80%) | 1 loaf | 3.9 | 750 g | 31 | 0 | 31 |
| 2 | 16 | Danish pastry (20%) |  | 3.9 | 3 pieces | 8 | 0 | 8 |
| 2 | 17 | Pastries, mixed (70%) | Arrak ball, shortcrust pastry filled w/almond dough glazed, | 9.6 | 3 pieces | 67 | 0 | 67 |
| 2 | 17 | Pizza, pirogue, frozen (30%) | Pizza (15%), 2 pieces  pirogue (15%), 2 pieces | 9.6 | 340 g  240 g | 29 | 0 | 29 |
|  |  |  |  |  |  |  |  | *185* |
|  |  | **Meat, meat products** |  |  |  |  |  |  |
| 3 | 22 | Beef, sirloin, fresh |  | 9.9 | 400-600 g | 99 | 10 | 89 |
| 3 | 23 | Pork chops, fresh |  | 15.2 | ~500 g | 152 | 15 | 129 |
| 3 | 24 | Lamb chops, fresh |  | 1 | 250-600 g | 10 | 30 | 7 |
| 3 | 25 | Chicken, whole, frozen |  | 14.9 | ~1 kg | 149 | 32 | 101 |
| 3 | 27 | Moose meat, frozen | 1 package | 1.7 | 240 g | 17 | 0 | 17 |
| 3 | 30 | Smoked ham (76%) | 1 package | 4.5 | 140-500 g | 34 | 0 | 34 |
| 3 | 30 | Bacon (24%) | 1 package | 4.5 | 120-140 g | 11 | 0 | 11 |
| 3 | 31 | Sausage, "Falu-type" (38%) |  | 19.9 | 600-800 g | 76 | 0 | 76 |
| 3 | 31 | Frankfurter (33%) |  | 19.9 | 300-900 g | 66 | 0 | 66 |
| 3 | 31 | Liver patty (18%) |  | 19.9 | 200 g | 36 | 0 | 36 |
| 3 | 31 | Mettwurst, smoked (11%) |  | 19.9 | 120-400 g | 22 | 0 | 22 |
| 3 | 34 | Meat soup (canned) | 1 jar | 0.6 | 500-600 g | 6 | 0 | 6 |
| 3 | 36 | Hamburgers (frozen)(60%) | 1 package | 16.5 | 400-600 g | 99 | 0 | 99 |
| 3 | 36 | Stuffed cabbage roll, frozen (24%) | 1 package (4 pieces) | 16.5 | 400 g | 40 | 0 | 40 |
| 3 | 36 | Swedish hashed potatoes with ham (16%) | 1 package | 16.5 | 1 kg | 26 | 0 | 26 |
|  |  |  |  |  |  |  |  | *759* |
|  |  | **Fish and seafood** |  |  |  |  |  |  |
| 4 | 38 | Plaice, filet, fresh | Frozen if missing | 0.6 | 300-400 g | 6 | 0 | 6 |
| 4 | 39 | Cod, filet, fresh | Frozen if missing | 0.6 | ~500 g | 6 | 0 | 6 |
| 4 | 40 | Baltic herring, filet, fresh | Frozen if missing | 1.1 | 300-500 g | 11 | 10 | 10 |
| 4 | 41 | Salmon, filet fresh | Frozen if missing | 2.0 | 300-500 g | 20 | 10 | 18 |
| 4 | 43 | Pike, perch, fresh (whole) | Pike | 1.0 | ~1 kg | 10 | 50 | 5 |
| 4 | 45 | Plaice, filet, frozen | 1 package | 3.8 | 300-400 g | 6 | 0 | 6 |
| 4 | 46 | Cod, filet, frozen | 1 package | 3.8 | 400 g | 17 | 0 | 17 |
| 4 | 47 | Mackerel, filet, frozen | 1 package | 3.8 | 400 g | 15 | 0 | 15 |
| 4 | 49-51 | Smoked mackerel/salmon |  | 1.3 | 300-350 g | 13 | 20 | 10 |
| 4 | 52 | Swedish caviar (spread) | 1 tube | 2.0 | 165-250 g | 20 | 0 | 20 |
| 4 | 53 | Pickled herring | 1 jar | 2.0 | 250 g | 20 | 50 | 10 |
| 4 | 54 | Canned tuna, in oil | 1 can | 1.3 | 185-200 g | 13 | 0 | 13 |
| 4 | 55-56 | Fish quenelles, canned (50%) | 1 can | 2.9 | 375 g | 15 | 0 | 15 |
| 4 | 55-56 | Fish fingers, frozen (50%) | 1 package | 2.9 | 230-900 g | 15 | 0 | 15 |
| 4 | 58 | Shrimp, unpeeled, frozen | 1 package | 1.5 | 400 g | 15 | 62 | 6 |
| 4 | 59 | Shrimps, canned | 1 can | 2.0 | 320-700 g | 20 | 30 | 14 |
|  |  |  |  |  |  |  |  | *185* |
|  |  | **Dairy products** |  |  |  |  |  |  |
| 5 | 61 | Milk, 0.5 % fat |  | 21.3 | 1-1.5 L | 213 | 0 | 213 |
| 5 | 62 | Milk, 1.5 % fat |  | 49.4 | 1 L | 494 | 0 | 494 |
| 5 | 63 | Milk, 3% fat |  | 25.9 | 1 L | 259 | 0 | 259 |
| 5 | 64 | Fermented milk, 0.5 % fat |  | 8.7 | 1 L | 29 | 0 | 29 |
| 5 | 66 | Yoghurt, 0.5 % fat |  | 8.7 | 1 L | 29 | 0 | 29 |
| 5 | 66 | Fruit yoghurt, 0.5 % fat |  | 8.7 | 1 L | 29 | 0 | 29 |
| 5 | 67 | Fermented milk, 1.5 % fat |  | 5.1 | 1 L | 51 | 0 | 51 |
| 5 | 65 | Fermented milk, 3 % fat |  | 18.6 | 1 L | 61 | 0 | 61 |
| 5 | 68 | Fruit yoghurt, >2 % fat (50 %) |  | 18.6 | 1 L | 61 | 0 | 61 |
| 5 | 68 | Plain yoghurt, 3 % fat (50 %) |  | 18.6 | 1 L | 61 | 0 | 61 |
| 5 | 70 | Cream, 12 % fat |  | 2.1 | 2-3 dL | 21 | 0 | 21 |
| 5 | 71 | Sour cream, 12 % fat |  | 1.6 | 3 dL | 16 | 0 | 16 |
| 5 | 72 | Whipping cream, 40 % fat |  | 5.5 | 3 dL | 55 | 0 | 55 |
| 5 | 75 | Cheese, 28 % fat (90%) |  | 12.6 | 0.5-1 kg | 113 | 0 | 113 |
| 5 | 75 | Cheese, 17 % fat (10%) |  | 12.6 | 0.5-2 kg | 13 | 0 | 13 |
| 5 | 76 | Cheese spread, 10 % fat | 1 package | 1.5 | ~400 g | 15 | 0 | 15 |
| 5 | 78 | Cottage cheese (75 %) |  | 3.7 | 250-500 g | 28 | 0 | 28 |
| 5 | 78 | Dessert cheese, Camenbert type (25 %) | 1 package | 3.7 | 125-150 g | 9 | 0 | 9 |
|  |  |  |  |  |  |  |  | *1557* |
|  |  |  |  |  |  |  |  |  |
| 6 | 80 | **Eggs** |  | 9.6 | 20-30 pc | 96 | 12 | *84* |
|  |  |  |  |  |  |  |  |  |
|  |  | **Fats** |  |  |  |  |  |  |
| 7 | 82 | Butter |  | 1.8 | 500 g | 18 | 0 | 18 |
| 7 | 83 | Baking margarine (33%) | 2 brands | 5.8 | 0.5-1 kg | 19 | 0 | 19 |
| 7 | 83 | Spread, 70-80 % fat (33%) | Butter-vegetable oil blend, 75% fat | 5.8 | 600 g | 19 | 0 | 19 |
| 7 | 83 | Spread, 70-80 % fat (3%) |  | 5.8 | 400 g | 2 | 0 | 2 |
| 7 | 83 | Liquid margarine, 80 % fat (16%) | 2 brands | 5.8 | 500 g | 9 | 0 | 9 |
| 7 | 83 | Spread, 60 % fat (10%) | Butter-vegetable oil blend, fat 60% | 5.8 | 600 g | 6 | 0 | 6 |
| 7 | 83 | Spread, 60 % fat (5%) | 2 brands | 5.8 | 600 g | 3 | 0 | 3 |
| 7 | 84 | Low-fat spread (85%) | 4 brands | 4.0 | 750 g | 34 | 0 | 34 |
| 7 | 84 | Low-fat spread (15%) | 1 brand, butter-oil blend | 4.0 | 750 g | 6 | 0 | 6 |
| 7 | 86 | Mayonnaise | 1 jar | 1.1 | 500 g | 11 | 0 | 11 |
| 7 | 87 | Rapeseed oil (55%) |  | 1.8 | 0.5-1 L | 10 | 0 | 10 |
| 7 | 87 | Olive oil (27%) |  | 1.8 | 0.5-1 L | 5 | 0 | 5 |
| 7 | 87 | Corn oil (18%) |  | 1.8 | 0.5-1 L | 3 | 0 | 3 |
|  |  |  |  |  |  | 145 |  | *145* |
|  |  | **Vegetables, roots** |  |  |  |  |  |  |
| 8 | 89 | Carrots |  | 7.8 | > 0.5 kg | 78 | 12 | 69 |
| 8 | 90 | Beetroots |  | 1.7 | > 0.5 kg | 17 | 20 | 14 |
| 8 | 92 | Cucumber |  | 5.1 | 1 piece | 51 | 5 | 48 |
| 8 | 93 | Brown onion |  | 7.5 | > 0.5 kg | 75 | 7 | 70 |
| 8 | 94 | Leek |  | 1.1 | 2-4 pieces | 11 | 16 | 9 |
| 8 | 95 | Cauliflower |  | 0.9 | 1 piece | 9 | 21 | 7 |
| 8 | 96 | White cabbage |  | 5 | 1 piece | 50 | 20 | 40 |
| 8 | 97 | Iceberg lettuce |  | 5.7 | 1 piece | 57 | 5 | 54 |
| 8 | 98 | Tomatoes |  | 10.1 | > 0.5 kg | 101 | 0 | 101 |
| 8 | 99 | Green pepper |  | 8.5 | 3 pieces | 85 | 15 | 72 |
| 8 | 101 | Green peas and carrots, frozen (79%) | 1 package | 5.5 | 1 kg | 43 | 0 | 43 |
| 8 | 101 | Spinach, frozen (21%) | 1 package | 5.5 | 0.45-1 kg | 12 | 0 | 12 |
| 8 | 102 | Yellow peas, dried |  | 0.7 | 0.5-1 kg | 7 | 0 | 7 |
| 8 | 103 | Pickled cucumber | 1 jar | 3.3 | 290-590 g | 33 | 33 | 22 |
| 8 | 104 | Canned peas and carrots (25%) | 1 can | 12.1 | 400-530 g | 30 | 33 | 20 |
| 8 | 104 | Canned mushrooms (25%) | 1 can | 12.1 | 190-400 g | 30 | 33 | 20 |
| 8 | 104 | Canned string beans (10%) | 1 can | 12.1 | 400 g | 12 | 33 | 8 |
| 8 | 104 | Canned tomatoes (40%) | 1 can/package | 12.1 | 400 g | 48 | 0 | 48 |
| 8 | 118 | Canned vegetable soup | 1 jar | 4 | 0.5-1 L | 40 | 0 | 40 |
|  |  |  |  |  |  |  |  | *704* |
|  |  | **Fruit and berries** |  |  |  |  |  |  |
| 9 | 106 | Oranges |  | 18.4 | ~1 kg | 184 | 29 | 131 |
| 9 | 107 | Grapes |  | 2.9 | 300-500 g | 12 | 4 | 11 |
| 9 | 108 | Hazelnuts |  | 2.5 | 200 g | 10 | 0 | 10 |
| 9 | 109 | Apples |  | 16 | 1 kg | 131 | 8 | 121 |
| 9 | 109 | Pears |  | 16 | 300-500 g | 29 | 8 | 26 |
| 9 | 110 | Peach, nectarine |  | 2.9 | 3 pieces | 29 | 13 | 25 |
| 9 | 111 | Bananas (80%) |  | 22.6 | ~1 kg | 194 | 37 | 122 |
| 9 | 111 | Melons (10%) |  | 22.6 | 1 piece | 23 | 48 | 12 |
| 9 | 111 | Kiwi fruit (10%) | 3 pieces | 22.6 | 400-800 g | 23 | 15 | 19 |
| 9 | 112 | Strawberries, fresh/frozen |  | 2.5 | 0.25-1 kg | 25 | 0 | 25 |
| 9 | 114 | Raisins |  | 1.3 | 200-500 g | 13 | 0 | 13 |
| 9 | 115 | Canned peaches | 1 can | 4.2 | 410-850 g | 42 | 0 | 42 |
| 9 | 116 | Lingonberry jam | 1 jar | 7.4 | 0.4-1.5 kg | 74 | 0 | 74 |
| 9 | 120 | Orange juice, concentrated (20%) |  | 23.6 | 2 dL | 47 | 0 | 47 |
| 9 | 120 | Orange juice, ready-to-drink (10%) |  | 23.6 | 1-1.5 L | 24 | 0 | 24 |
| 9 | 120 | Apple juice, concentrated (10%) |  | 23.6 | 2 dL | 24 | 0 | 24 |
| 9 | 120 | Cordial ready-to-drink (15%) | 3 types | 23.6 | 7.5 dL | 35 | 0 | 35 |
| 9 | 120 | Cordial, concentrated, mixed fruit/berries (45%) | 1 bottle | 23.6 | 1-1.5 L | 106 | 0 | 106 |
|  |  |  |  |  |  |  |  | *867* |
|  |  | **Potatoes** |  |  |  |  |  |  |
| 10 | 122 | Potatoes |  | 44.9 | 2 kg | 449 | 22 | 350 |
| 10 | 123 | Mashed potato powder | 1 package | 0.5 | 500-855 g | 5 | 0 | 5 |
| 10 | 125 | French fries, frozen | 1 package | 8.7 | 0.9-2 kg | 87 | 0 | 87 |
| 10 | 127 | Potato crisps | 1 bag | 1.6 | 200-400 g | 16 | 0 | 16 |
|  |  |  |  |  |  |  |  | *458* |
|  |  | **Sweets, sugar, ice craem** |  |  |  |  |  |  |
| 11 | 130 | Caster sugar |  | 6.4 | 1-2 kg | 64 | 0 | 64 |
| 11 | 137 | Instant chocolate drink powder | 1-2 brands | 2.1 | 0.5-1 kg | 21 | 0 | 21 |
| 11 | 139 | Honey | 1 jar | 0.7 | 350-700 g | 7 | 0 | 7 |
| 11 | 140 | Chocolate, assorted (51%) | 1-2 brands | 15.2 | 500g | 78 | 0 | 78 |
| 11 | 140 | Candy, mixed (49%) | assorted | 15.2 | 400-500 g | 74 | 0 | 74 |
| 11 | 141 | Ketchup (80%) | 1 package | 13.6 | 0.5-1 kg | 109 | 0 | 109 |
| 11 | 141 | Sauce Bearnaise/Hollandaise (10%) | 1 package | 13.6 | 500 mL | 14 | 0 | 14 |
| 11 | 141 | Salad dressing (10%) |  | 13.6 | 260-300 mL | 14 | 0 | 14 |
| 11 | 142 | Ice cream, tub, 10 % fat (50 %) | 3-4 brands | 11.3 | 1.5-6 L | 28 | 0 | 28 |
| 11 | 142 | Ice cream, cone/stick (50 %) | 2 brands | 11.3 | 2 pieces | 28 | 0 | 28 |
| 11 | 145 | Mustard | 1 package | 1.9 | 500 mL | 16 | 0 | 16 |
|  |  |  |  |  |  |  |  | *453* |
|  |  | **Beverages** |  |  |  |  |  |  |
| 12 | 148 | Soda (90%) | 3 varieties | 87.7 | 3 cans | 789 | 0 | 789 |
| 12 | 148 | Soda, light (10%) | 3 varieties | 87.7 | 3 cans | 88 | 0 | 88 |
| 12 | 149 | Mineral water | 2 brands | 10.8 | 2 cans | 108 | 0 | 108 |
| 12 | 151 | Beer, low-alcohol <2.2 % | 3 brands | 4.5 | 3 cans | 45 | 0 | 45 |
| 12 | 152 | Beer, 2.8 % alcohol | 3 brands | 17.5 | 3 cans | 175 | 0 | 175 |
|  |  |  |  |  |  |  |  | *1205* |

Web Table 2. Mean content of individual fatty acids in MB analyzed in 2005 and 2010.

g/pers/day.

| Fatty acid | 2005 | 2010 |
| --- | --- | --- |
| C 4:0 | 1.35 | 0.76 |
| C 6:0 | 0.75 | 0.44 |
| C 8:0 | 0.64 | 0.60 |
| C 10:0 | 1.12 | 1.11 |
| C 12:0 | 3.40 | 3.89 |
| C 13:0 | 0.02 | 0.02 |
| C 14:0 | 4.81 | 4.98 |
| C 15:0 | 0.36 | 0.35 |
| C 15:0 ai | 0.15 | 0.12 |
| C 15:0 i | 0.09 | 0.08 |
| C 16:0 | 23.9 | 24.9 |
| C 16:0 ai | n.d. | n.d. |
| C 16:0 i | 0.06 | 0.01 |
| C 17:0 | 0.29 | 0.30 |
| C 17:0 ai | 0.20 | 0.16 |
| C 17:0 i | 0.17 | 0.15 |
| C 18:0 | 8.31 | 9.84 |
| C 18:0 ai | n.d. | n.d. |
| C 18:0 i | 0.02 | 0.00 |
| C 20:0 | 0.31 | 0.39 |
| C 22:0 | 0.18 | 0.19 |
| C 23:0 | n.d. | n.d. |
| C 24:0 | 0.10 | 0.06 |
| C 14:1 | 0.34 | 0.31 |
| C 15:1 | n.d. | n.d. |
| C 16:1 | 1.47 | 1.22 |
| C 17:1 | 0.21 | 0.19 |
| C 18:1 | 36.0 | 40.3 |
| C 20:1 | 0.71 | 0.76 |
| C 22:1 | 0.29 | 0.47 |
| C 24:1 n-9 | 0.07 | 0.05 |
| C 16:2 n-4 | 0.01 | 0.02 |
| C 16:3 | 0.01 | 0.00 |
| C 16:4 n-3 | 0.01 | 0.01 |
| C 18:2, total | 10.90 | 12.0 |
| C 18:2 cis n-6 | 10.19 | 11.6 |
| C 18:2 konj | 0.22 | 0.18 |
| C 18:3 n-3 | 2.53 | 2.59 |
| C 18:3 n-6 | 0.02 | 0.01 |
| C 18:4 n-3 | 0.04 | 0.08 |
| C 20:2 n-6 | 0.07 | 0.05 |
| C 20:3 n-3 | 0.02 | 0.02 |
| C 20:3 n-6 | 0.05 | 0.04 |
| C 20:4 n-3 | 0.02 | 0.03 |
| C 20:4 n-6 | 0.13 | 0.12 |
| C 20:5 n-3 | 0.14 | 0.18 |
| C 21:5 n-3 | n.d. | 0.01 |
| C 22:2 n-6 | n.d. | n.d. |
| C 22:4 n-3 | n.d. | n.d. |
| C 22:4 n-6 | n.d. | 0.01 |
| C 22:5 n-3 | 0.04 | 0.06 |
| C 22:5 n-6 | n.d. | 0.01 |
| C 22:6 n-3 | 0.23 | 0.33 |
| C 14:1t | 0.14 | 0.10 |
| C 16:1t | 0.28 | 0.15 |
| C 18:1t | 1.26 | 1.03 |
| C 18:2t | 0.11 | 0.33 |
| C 18:3t | 0.06 | 0.11 |
| Trans, total | 1.85 | 1.72 |
| Other | 0.13 | 0.13 |
| *Rest* | 0.45 | 0.49 |
|  |  |  |
| SFA | 46.2 | 48.4 |
| MUFA | 39.1 | 43.3 |
| PUFA | 14.2 | 15.5 |
| n-6, total | 11.2 | 12.2 |
| n-3, total | 3.0 | 3.3 |

n.d. = not detected
